# Transcutaneous Spinal Cord Stimulation to Stabilize Seated Systolic Blood Pressure in Persons With Chronic Spinal Cord Injury: Protocol Development

**DOI:** 10.1089/neur.2023.0063

**Published:** 2023-12-19

**Authors:** Caitlyn G. Peters, Noam Y. Harel, Joseph P. Weir, Yu-Kuang Wu, Lynda M. Murray, Jorge Chavez, Fiona E. Fox, Christopher P. Cardozo, Jill M. Wecht

**Affiliations:** ^1^ James J Peters VA Medical Center, Bronx, New York, USA.; ^2^ Kessler Foundation, West Orange, New Jersey, USA.; ^3^ Icahn School of Medicine at Mount Sinai, New York, New York, USA.; ^4^ University of Kansas, Lawrence, Kansas, USA.

**Keywords:** autonomic nervous system, blood pressure, electrical stimulation, methodology, spinal cord injury

## Abstract

Transcutaneous spinal cord stimulation (tSCS) is an emerging therapeutic strategy to target spinal autonomic circuitry to normalize and stabilize blood pressure (BP) in hypotensive persons living with chronic spinal cord injury (SCI). Our aim is to describe our current methodological approach to identify individual tSCS parameters that result in the maintenance of seated systolic blood pressure (SBP) within a pre-defined target range. The parent study is a prospective, randomized clinical trial in which eligible participants will undergo multiple mapping sessions to optimize tSCS parameter settings to promote stable SBP within a target range of 110–120 mm Hg for males and 100–120 mm Hg for females. Parameter mapping includes cathode electrode placement site (T7/8, T9/10, T11/12, and L1/2), stimulation frequency (30, 60 Hz), current amplitudes (0–120 mA), waveform (mono- and biphasic), pulse width (1000 μs), and use of carrier frequency (0, 10 kHz). Each participant will undergo up to 10 mapping sessions involving different electrode placement sites and parameter settings. BP will be continuously monitored throughout each mapping session. Stimulation amplitude (mA) will be increased at intervals of between 2 and 10 mA until one of the following occurs: 1) seated SBP reaches the target range; 2) tSCS intensity reaches 120 mA; or 3) the participant requests to stop. Secondary outcomes recorded include 1) symptoms related to autonomic dysreflexia and orthostatic hypotension, 2) Likert pain scale, and 3) skin appearance after removal of the tSCS electrode.

Clinical Trials Registration: NCT05180227

## Introduction

Spinal cord injury (SCI) disrupts crucial communication between the spinal cord circuitry and supraspinal control centers, resulting in numerous sensorimotor and autonomic impairments.^1^ In persons with SCI, impaired autonomic nervous system (ANS) function gives rise to persistent hypotension and bradycardia, with episodic falls in blood pressure (BP) during orthostatic repositioning (orthostatic hypotension; OH) and severe increases in BP during autonomic dysreflexia (AD). Unmitigated hypotension and OH are associated with cognitive deficits,^2–5^ decrease in exercise performance,^6^ and adverse changes in health-related quality of life.^7,8^ There is also an increased risk of stroke^9^ and early-onset dementia and Alzheimer's disease in persons with SCI compared to uninjured controls,^10,11^ which has been associated with impaired cerebrovascular regulation and buffering of BP attributable to loss of integral autonomic hemodynamic regulation.^12^ Hemodynamic impairments in persons with an SCI above T6 can adversely impact rehabilitation goals, re-employment, and reintegration into society after injury^13^; this may be why persons with SCI report restoration of autonomic functions as a higher priority than regaining the ability to walk.^14,15^

Treatment of hypotension and OH has generally focused on ameliorating symptoms of cerebral hypoperfusion, rather than normalizing BP, despite increasing documentation of severe BP abnormalities post-SCI.^16^ Current management of OH begins with non-pharmacological physical interventions, which include increases in salt and water intake to expand extracellular fluid volume, compression stockings or abdominal binders to limit extravascular fluid shift, and lower extremity functional electric stimulation to promote skeletal muscle pump action.^17^ Safety and efficacy of anti-hypotensive pharmacological agents have been tested in small-scale clinical trials in hypotensive persons with SCI, and the results indicate wide variation in individual response.

Examples of pharmacological treatments include droxidopa which is a synthetic amino acid that is converted to norepinephrine in neuronal and non-neuronal tissue,^17^ fludrocortisone which is a mineralocorticoid that increases blood volume,^17^ ephedrine which is a non-selective alpha- and beta-receptor agonist with central and peripheral effects,^17^ nitro-L-arginine methyl ester, a nitric oxide synthase inhibitor, which has been shown to induce large increases in systemic BP in persons with tetraplegia,^17^ and midodrine hydrochloride, a selective alpha-agonist that binds to and activates adrenergic receptors in the arterial and venous vasculature, causing increases in vascular tone and BP.^17^

Alarmingly, we recently reported increased BP instability after the administration of a single dose of midodrine (10 mg), which is the most commonly prescribed antihypotensive medication for persons with SCI.^18^ Further, after 30 days of midodrine administration, persons with SCI had increased fluctuations in BP, no improvement in symptoms of OH, and significantly worsened intensity of symptoms reported in association with AD; this severely limits the utility of midodrine for treatment of OH in the SCI population. As such, alternate methods of stabilizing BP within a normotensive range must be explored.

Electrical spinal cord stimulation (SCS) has emerged as a potentially promising treatment that improves ANS function to increase and stabilize BP post-SCI.^19^ Epidural SCS (eSCS) involves the implantation of a 16-electrode array into the epidural space, usually in the lumbar or lower thoracic region of the spinal cord. A range of studies have attempted to define the optimal eSCS locations and stimulation pattern settings for restoring BP in animal and human models of SCI. In a pre-clinical study, Squair and colleagues demonstrated that eSCS mapping at the T11–T13 spinal segments in an animal model of SCI showed the most robust increases in BP, correlating with a high density of sympathetic pre-ganglionic neurons at those spinal segments.^20^ The same laboratory demonstrated that eSCS at T11–T13 was able to mitigate the fall in BP during an orthostatic provocation, using a lower body negative-pressure chamber, in a rodent model of T3 contusion injury.^20^

Additional clinical studies support the effect of restoring BP function to facilitate better orthostatic BP control in persons with chronic SCI. A case report demonstrated that eSCS at T10–T11 spinal segments increased SBP, plasma norepinephrine, and peroneal nerve muscle sympathetic nerve activity in a person with complete cervical SCI.^20^ During an orthostatic provocation, several reports demonstrate that using eSCS at sites including T11–L1 vertebral levels effectively mitigates the fall in BP during a head-up tilt,^219,21–23^ and improves cerebral blood flow velocity,^23^ cardiac stroke volume, and cardiac contractility,^22,23^ thereby eliminating symptoms of orthostatic intolerance^19,23^ in persons with SCI. Moreover, daily 2-h sessions of lumbosacral eSCS over 80 sessions alleviated the fall in BP during a sit-up test without the use of active stimulation in 4 hypotensive persons with chronic, cervical, motor-complete SCI.^24^

These results suggest that targeted SCS may be associated with adaptive plasticity that restores intrinsic cardiovascular regulatory function. Parameter settings included in these published eSCS reports suggest that the optimal epidural electrode placement site for orthostatic BP control varies among the small cohort of participants studied to date from spinal segments T10–L1, cathode: 0–1, 11–12 and anode: 5–6 (*n* = 1),^20^ T10–L2, cathode: 1, 4, 6–10, 12, and 15 and anode: 0, 2–3, 5, 11, and 13–15; (*n* = 1),^23^ L1–S1, three studies where stimulation parameters were individualized for each participant (*n* = 7,^21^ *n* = 4,^19^ and *n* = 4^24^), and L2–S2, cathode placement for participant 1: 0, 6, and 11 and anode placement for participant 1: 4, 10, and 15; cathode for participant 2: 0, 5, and 11 and anode for participant 2: 4, 9, and 15 (*n* = 2).^22^ Moreover, stimulation parameters vary on an individual basis, with frequencies ranging between 15 and 120 Hz, pulse width between 300 and 500 μs, and intensity amplitudes between 2 and 15 V, which were generally increased until BP was normalized.

Samejima and colleagues^1^ summarized the most plausible mechanisms underlying the effects of neuromodulation on ANS function in persons with SCI. Electrical stimulation of the somatoautonomic reflex through primary afferent fibers proximal to the spinal segments near the stimulation electrode may lead to the excitation of spinal interneurons that activate autonomic efferent pathways consisting of sympathetic pre- and post-ganglionic neurons. The somatoautonomic reflex has been defined as a reflex elicited by stimulation of somatic tissue, manifesting as an alteration of ANS activity.^25^ Targeted ANS excitation may lead to appropriate modulation of target organ function,^1^ such as peripheral vascular vasoconstriction during changes in orthostatic positioning. Additionally, chronic SCS may induce axon sprouting, myelin preservation, and remyelination around the lesion site to promote neuroplastic changes within the spinal autonomic circuits, which has been noted in rodent models of SCI.^1,26,27^ However, concrete mechanistic evidence is still lacking to support these hypotheses.

In addition to eSCS, transcutaneous SCS (tSCS) has been used as a neuroprosthetic to promote ANS recovery in persons with chronic SCI.^28^ As a non-invasive approach, tSCS is theorized to target dorsal afferents and excite inter- and intrasegmental neurons resulting in depolarization of sympathetic pre-ganglionic neurons that facilitate increases in vascular tone.^28^ The protocol reported by Phillips and colleagues involved the use of a self-adhesive cathode electrode (∼30 mm) placed at the interspinous process of the thoracic-7/8 vertebral level and anodes (5 × 9 cm) placed symmetrically over the right and left iliac crests, using a monophasic waveform, 1-msec pulse duration, 30 Hz, with tSCS amplitudes ranging from 10 to 70 mA. Using this tSCS strategy, Phillips and colleagues demonstrated improvement in orthostatic hemodynamics, which corresponded to improved cardiac contractility and increased cerebral blood flow velocity during a head-up tilt maneuver in 5 persons with chronic, motor-complete SCI.^28^

Interestingly, eSCS (T10–L1 vertebral levels; 17–35 Hz; 300–500 us, 4.0–6.8 V)^29^ and tSCS (T7/8; 30 Hz, biphasic, 2-ms pulse width, 20–30 mA)^30^ have been shown to mitigate increases in BP during AD and prevent AD-induced BP elevations by bladder distention^29^ and digital anal rectal stimulation^30^ in persons with chronic, motor-complete SCI. This suggests that either form of SCS may be a viable clinical option to eliminate ANS deficits and restore homeostatic control of BP. However, given the wide range of stimulus locations, frequencies, pulse widths, and waveforms applied across studies, it remains to be determined whether a single optimal set of parameters exists or whether, more likely, parameter settings will need to be individually optimized to stabilize SBP. Moreover, before SCS can be implemented as a widespread clinical intervention to promote stable BP, a systematic mapping protocol must be identified that enables the accurate, reliable, and reproducible identification of individualized optimal stimulation settings for BP control.

tSCS may be a new non-invasive, non-pharmacological, therapeutic option to target spinal autonomic circuitry to rapidly normalize and stabilize BP in hypotensive persons with SCI. Therefore, this article aims to describe our current mapping methodology using tSCS location, baseline and carrier frequency, waveform, pulse width, and stimulation amplitude to maintain seated BP within a pre-defined target range in persons with chronic, hypotensive SCI at and above T6.

## Methods and Analyses

### Participant demographics

The parent study is an open-label, prospective, randomized clinical trial in which 10 participants with SCI will be identified at the James J. Peters VA Medical Center (JJP VAMC) through a variety of recruitment strategies including: 1) flyers which will be distributed at the hospital and local community venues, 2) past study participants having signed a Notice of Privacy Practices and agreed to be informed about new research, 3) physician referral, and 4) person's request. Pre-enrollment criteria include persons with chronic SCI at T6 and above, at least 1 year post-injury. Based on the International Standards for Neurological Classification of Spinal Cord Injury, we will recruit persons with grade A, B, or C who are non-ambulatory (i.e., wheelchair-bound) and non-ventilatory dependent. The exclusion criteria are outlined (Table 1). The definition of hypotension varies in the SCI population. The recent International Standards to document Autonomic Function following SCI defines hypotension as supine systolic blood pressure (SBP) <90 mm Hg or supine diastolic pressure <60 mm Hg^31^; however, in 1978, the World Health Organization (WHO) defined hypotension as SBP ≤110 mm Hg for males or ≤100 mm Hg for females.^32^

**Table 1.** Participant Inclusion and Exclusion Criteria

Inclusion criteria	Exclusion criteria
1. Adults ≥18 years of age	1. Current illness or infection
2. Traumatic SCI lesions T6 and above	2. Controlled or uncontrolled diabetes mellitus or hypertension
3. American Spinal Injury Association impairment scales A, B, or C	3. Currently taking antihypertensive medication
4. Non-ambulatory	4. Neurological condition other than SCI (Alzheimer's disease, multiple sclerosis, or Parkinson's disease)
5. Non-ventilatory dependent	5. History of cardiovascular disease (coronary artery disease, congestive heart failure, or peripheral artery disease)
6. Hypotensive (SBP ≤110 mm Hg for males or ≤100 mm Hg for females)	6. Present or history of thrombosis in the past 12 months
	7. Severe contractures

SCI, spinal cord injury; SBP, systolic blood pressure.

**FIG. 1.**
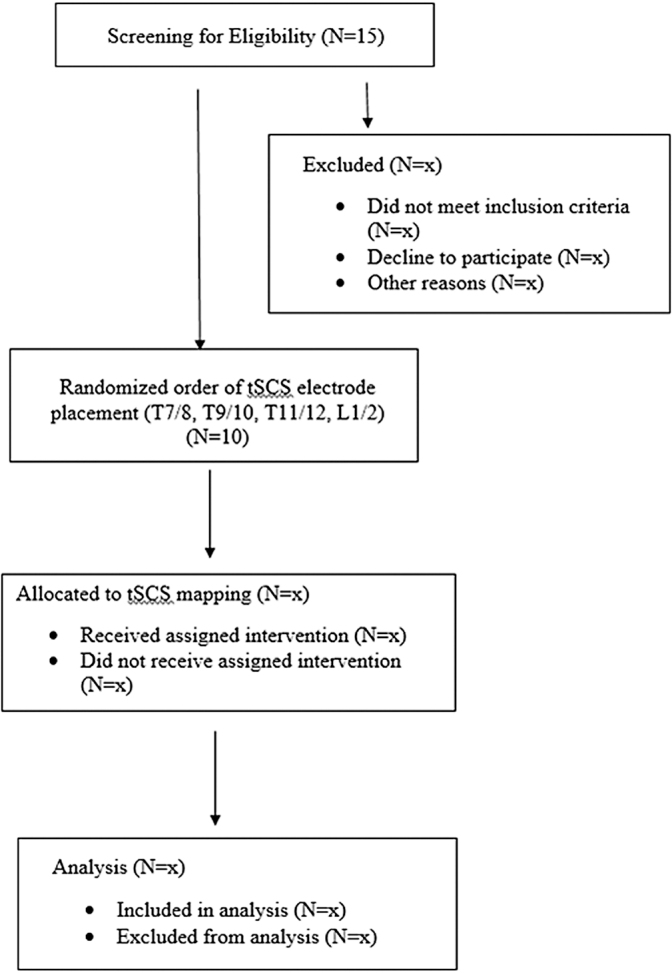
Flow diagram of enrollment, screening, and assessments. tSCS, transcutaneous spinal cord stimulation.

In addition, we have shown adverse effects of seated SBP below these thresholds on cognitive function and cerebral blood flow in persons with SCI (average seated SBP in tetraplegia group: 99 ± 18 mm Hg).^4^ Our initial screening visit will be conducted to determine hypotensive status defined by the WHO. A flow diagram of enrollment, screening, and assessments is provided (Fig. 1). Participants will have flexibility in scheduling appointments and receive compensation per study visit to help with participant retention.

### Ethics and dissemination

The experimental protocol is registered on clinical trials.gov (NCT05180227) and was approved by the JJP VAMC Institutional Review Board on March 15, 2022. All participants will undergo an informed consent process with the Principal Investigator or other study personnel before enrollment in accordance with the 1964 Declaration of Helsinki. This project has received U.S. Food and Drug Administration investigational device exemption approval. Interim analysis will be conducted with the intent to disseminate preliminary findings to be used by other groups to allow for more seamless interpretation of the fundings based on a similar methodological approach to the application of tSCS in the SCI population. Trial results will be disseminated through peer-reviewed publications, conference presentations, and seminars.

### Patient and public involvement

Patients or the public will not be involved in the design, conduct, reporting, or dissemination plans of our research.

### Randomization

Each participant will complete up to 10 visits, with two different mapping sessions per visit, and at least 3 days' washout between visits. For the first mapping site on each visit, the tSCS cathodal site (interspinous space between T7/8, T9/10, T11/12, and L1/2) will be randomly assigned using a matrix created with Research Randomizer barring a contraindication at the selected site such as a scar or open wound. The second site assigned at each visit will be two segments above or below the first site to avoid skin irritation from consecutive cathode placement sites (e.g., first site selected: T7/8; second location: T11/12). Each visit will be conducted in ∼2 h with ample time between the two mapping sessions to allow the BP to return to baseline, the participant to weight-shift for pressure relief, and bladder voiding if needed.

### Transcutaneous electrical spinal cord stimulation intervention

The tSCS stimulator (Digitimer DS8R Constant Current Stimulator; Digitimer Ltd, Welwyn Garden City, UK) can be configured to generate different waveforms (bi- and monophasic), pulse duration, stimulation amplitudes, and pulse widths. A microcontroller circuit board (Arduino Uno Rev3; Arduino S.r.l, Genova, Italy), and Integrated Development Environment software will be programmed to communicate with the stimulator (DS8R) regarding the use (or not) of a carrier frequency (10 kHz) and to set and manipulate pulse width and stimulation frequency. The self-adhesive cathode electrode used is a ∼2.5-cm round electrode (PALS; Axelgaard Manufacturing Co., Ltd, Fallbrook, CA) and two 5 × 10 cm self-adhesive electrodes (ValueTrode; Axelgaard Manufacturing Co., Ltd) located symmetrically on the skin over the iliac crests as anodes. A custom LabVIEW program will be designed to manipulate current intensity (mA) in real time while simultaneously collecting beat-to-beat changes in BP, heart rate (HR), respiratory rate (Rr), and stimulation amplitude by a 12-bit analog-to-digital converter (DAQcard- USB-6221; National Instruments, Austin, TX). Participants, but not investigators, will be blinded to the cathode placement site and stimulus parameters.

### Assessments

Experiments will be conducted in a quiet, dimly lit, and temperature-controlled room (72°F), and participants will be instructed to avoid consuming caffeine, alcohol, and nicotine products for the 12 h before testing. We will ask that participants refrain from vigorous exercise for 24 h preceding arrival, and upon arrival, they will be asked to empty their bladder. All mapping sessions will be completed in a seated position with participants in their wheelchairs.

Before testing, baseline seated BP will be assessed for no less than 10 min to ensure a hypotensive state. Before beginning the tSCS amplitude ramp, the skin will be checked at and around the cathode placement site for any scarring, moles, tags, or redness, and a picture will be captured for comparison with post-testing. After the first cathode site is tested, the skin will be checked for any changes in skin integrity or redness and a second picture will be captured and stored. Before beginning the second cathode electrode amplitude ramp, we will allow ample time (10+ min) for seated BP to return to baseline.

During each amplitude ramp, the other tSCS parameters will be maintained as follows: 1) frequency of either 30 or 60 Hz; 2) the waveform selected as either monophasic (not charged balanced) or biphasic; 3) the pulse width will be set to 1000 μs (1000 μs monopolar, or 500 μs per phase of biphasic, or burst duration of 1000 μs when carrier frequency is used); and 4) we will set the carrier frequency at either 0 or 10 kHz—for monophasic carrier frequency, 10 bursts of 70-μs pulses is delivered over 1000 μs; for biphasic carrier frequency, 10 bursts of 80-μs pulses (40 μs per phase) is delivered over 1000 μs. The tSCS combinations will be specified for each individual participant based on the SBP response and pain tolerance during the current and previous mapping sessions. Stimulation current (mA) will be ramped from 0 to as high as 120 mA based on seated SBP and participant tolerance. Ramping will be stopped when SBP reaches our target range: between 110 and 120 mm Hg for males and between 100 and 120 mm Hg for females; or if we reach 120 mA with no SBP response; or if the participant requests to stop. This trial is consistent with the Standard Protocol Items: Recommendations for Interventional Trial guidelines.^33^

### Primary outcome

#### Systolic blood pressure

**FIG. 2.**
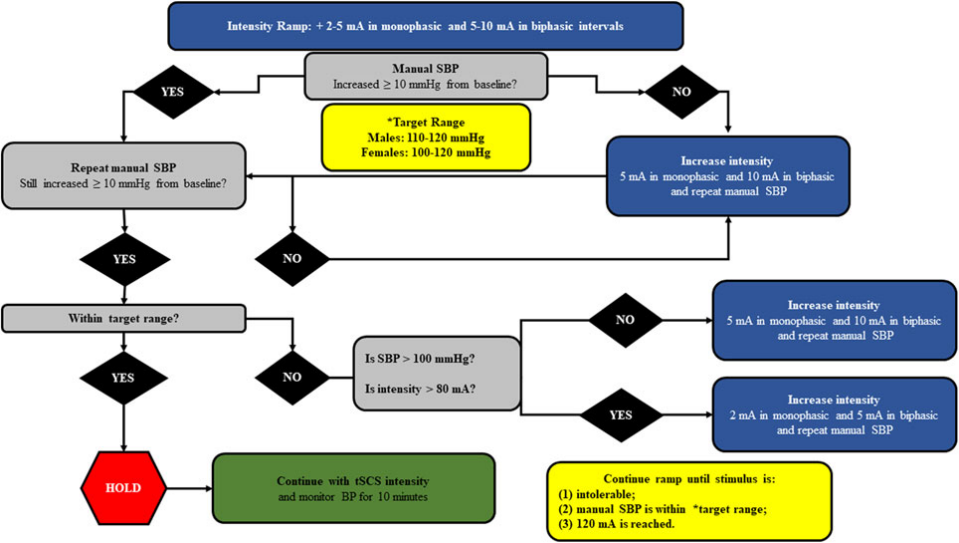
The decision-making algorithm for the mA intensity ramping protocol. SBP, systolic blood pressure; tSCS, transcutaneous spinal cord stimulation.

Each stimulation intensity ramp-interval will be held for at least ∼1 min to measure and record brachial BP with automatic auscultation (Root with non-invasive BP; Masimo, Irvine, CA) with continuous viewing of beat-to-beat BP (Finometer PRO; Finapres Medical Systems B.V., Enschede, The Netherlands). Our decision-making algorithm is presented (Fig. 2). Once the SBP reaches our target range (between 110 and 120 mm Hg for males and between 100 and 120 mm Hg for females), we will record seated hemodynamics for 10 min whereas the tSCS parameter settings remain constant. Specific outcome measures at each time point are presented (Table 2). If we have not observed an adequate or optimal increase in SBP within 30 min, we have conservatively decided to stop tSCS mapping because of the possibility of heating/burning the skin and allow participants time to relieve pressure and empty their bladder if needed.

**Table 2.** Summary of Assessments at Each Time Period

Study period		
	Enrollment	Mapping visits
Informed consent	X	
Medical history	X	
Eligibility screen for hypotension	X	
**Primary outcome**		
tSCS		X
Electrode placement site		X
(T7/8, T9/10, T11/12, L1/2)		
Frequency (30, 60 Hz)		X
Waveform (monophasic, biphasic)		X
Pulse width		X
Carrier frequency (10 kHz or none)		X
Blood Pressure		X
**Secondary outcomes**		
AD survey		X
Pain scale		X
Skin appearance		X

tSCS, transcutaneous spinal cord stimulation; AD, autonomic dysreflexia.

### Electrical stimulation characteristics

tSCS parameters will vary with every mapping session in each participant based on previous SBP response(s) to tSCS. Burst frequencies will include 30 Hz, which has been previously cited,^28^ and 60 Hz, as anecdotally reported to be effective in other laboratories. Pulse width is 1000 μs, delivered as either mono- or biphasic, with or without a 10-kHz carrier frequency as detailed earlier. Increasing pulse width has been shown to lead to greater fiber recruitment, which may increase distribution and sensation of paresthesia.^34,35^ Use of a high carrier frequency (10 kHz) has been described as providing inhibition of superficial nociceptive afferents, enabling the use of higher stimulation amplitudes with less pain in intact persons.^36–38^ We will compare BP responses to tSCS with and without the use of the most used high-frequency carrier (10 kHz). Delivered current amplitudes will be increased until effects on seated SBP are observed (target range as described above) or one of the criteria for discontinuing the ramp-up of current is reached.

### Systemic hemodynamics

We will use a bioamplifier (Model RESP 1 with electrocardiogram [ECG]; UFI, Morro Bay, CA), for continuous HR and Rr monitoring, with electrodes placed at the right and left mid-axillary lines in the fifth intercostal space and at the right anterior axillary line. Continuous beat-to-beat BP will be assessed at the finger using photoplethysmography, and brachial BP will be monitored at 1-min intervals with automatic auscultation. Beat-to-beat ECG and photoplethysmography BP signals will be sampled at 500 Hz and will be continuously monitored in real time on a computer screen. Systemic hemodynamics will be collected for 5 min in the seated baseline before the application of tSCS and then continuously throughout each mapping session. We will continue to monitor beat-to-beat hemodynamics after stimulation is turned off until BP is returned to ±10/5 mm Hg of baseline. Once the SBP reaches our target range, we will record seated hemodynamics for 10 min whereas the tSCS parameters remain constant. The digitized signals will be stored offline for subsequent analysis using custom data analysis programs written with LabVIEW graphical software for instrumentation (National Instruments, Austin, TX).

### Secondary outcomes

#### Tracking safety

Reporting safety and tolerability outcomes and adverse events are also critical to establishing a standardized methodology. During the mapping sessions, after each incremental increase in current amplitude, participants will be asked to report any change in symptoms, including tingling, tightness, paresthesia, muscle twitching, pins and needles, stabbing, and burning; however, we expect many persons to give different descriptors for the sensations experienced with tSCS and will ask open-ended questions. Although we expect most of the sensations to occur at or around the cathode electrode placement site, of which they are blinded, some may report feelings in the hands and feet, at the anode placement, and in the head. In addition, we will record perceived differences in disposition, including alertness, warmer, pressure, comfort, and breathing. Participants will be asked to describe tolerability and pain levels using a numerical Likert rating scale of 0 (none) to 10 (severe).

In addition, participants will be asked to rate the symptoms of AD (blurred vision, pounding headache, goosebumps, heart palpitations, sweating, ringing in the ears, and nausea) and OH (dizziness, lightheadedness, blurred vision, nausea, and syncope) using a numerical rating scale of 0 (none) to 10 (severe) during baseline and every 5 min during the mapping sessions. Finally, skin appearance will be assessed before and after electrode placement removal by description and photography (redness, discoloration, skin tear, burn, and scab). We intend to limit skin reactions by placing the electrode evenly on the skin, with equal pressure on the entire electrode surface, and will use medical gauze and tape to secure the electrode and limit skin pinching.

### Statistical analysis

The primary outcome variable will be SBP with secondary variables including indices of safety/tolerability (e.g., AD symptoms and pain). To examine whether there are consistent stimulation parameters that increase SBP, data will be analyzed using a mixed-modeling approach with participant modeled as a random effect and the following stimulation characteristics modeled as fixed effects: cathodal stimulation site (T7/8, T9/10, T11/12, and L1/2); stimulation frequency (30, 60 Hz); waveform (monophasic, biphasic); pulse width (1000 μs); carrier frequency (0, 10 kHz); and stimulation current (0–120 mA).

In addition, to quantify the effect of the tSCS, the optimal setting for each participant (based upon the BP response) will be compared to the baseline BP, from which the effect size (Cohen's *d*) and confidence interval will be calculated. All analyses will be performed using R software. The lme4 package will be used to perform the mixed-models analyses for the ratio-scale data. The ordinal package in R will be used to analyze the Likert-scale–type data. The 95% level of confidence will be used. We used the R software simr package for multi-level models for power calculations with fixed-effects variables of waveform and electrode site. Given the number of repeated measurements of SBP in each participant, we anticipate having ∼95% power to detect <2-mm-Hg mean differences at the 95% level of confidence.

## Discussion

This trial is focused on defining the tSCS protocol that can be used to identify individualized optimal stimulation parameter settings that safely and effectively increase and stabilize seated SBP in hypotensive persons with SCI. The knowledge gained from this study will begin to establish a standard methodology for individualized tSCS mapping of spinal neural circuits to promote the restoration of intrinsic ANS control of BP. Standardizing the methodology will allow for comparisons among research laboratories, to facilitate more widespread application of tSCS as a viable treatment for cardiovascular autonomic deficits post-SCI. Standardize methodologies for tSCS application to restore ANS function in both research and clinical settings will ultimately improve engagement in rehabilitation and daily activities to promote independence, vitality, and quality of life.

In future clinical trials, validation of surface landmarks used for electrode placement in the thoracic and lumbar vertebral regions will be imaged using lateral spine dual energy x-ray absorptiometry scans similar to methods previously published by our group.^39^ Once the electrodes are placed on each palpated vertebral spinous process, the lateral vertebral spine image will be used to validate the accurate placement of the electrode by locating the metal tab from the electrode.

### Limitations

We recognize that there are many more combinations of tSCS parameters than the ones we suggested in this report, but chose these parameters based on scant empirical evidence and acknowledge that additional research may be needed to more fully understand the best practice for the use of tSCS to increase and stabilize seated BP in persons with SCI.

Skeletal muscle contraction induced by tSCS could theoretically contribute to increased BP through compression of the venous vasculature of the lower extremity or splanchnic beds, thereby increasing venous return to the heart. Electromyography (EMG) of the trunk and lower limb skeletal muscles will not be recorded during the mapping session because of stimulation artifact; however, we plan to visualize trunk and lower extremity changes with active stimulation and visual relaxation of the muscles after stimulation is turned off. Future EMG recordings of skeletal muscle contractions should include the rectus abdominus, internal and external obliques, vastus lateralis, rectus femoris, medial hamstrings, tibialis anterior, soleus, and medial gastrocnemius.

## Conclusion

tSCS is a promising non-pharmacological treatment that may support intrinsic ANS regulatory function to facilitate BP control post-SCI. It has been postulated that the stimulation directly activates primary afferents leading to activation of pre-ganglionic sympathetic neurons. Previous studies have shown the feasibility of using tSCS to increase BP in small numbers of hypotensive persons with SCI; however, there is a lack of documentation and variability across equipment and parameters describing the methodology. Standardizing the methodology will allow for comparisons among investigations and will help validate tSCS as a viable treatment to increase and stabilize BP post-SCI, which will improve engagement in rehabilitation and daily activities to promote independence, vitality, and quality of life.

## Supplementary Material

Supplemental data

Supplemental data

## Data and Safety Monitoring

Physical study materials with identifying information will be kept on-site in secured rooms and cabinets, and electronic study materials will be kept in a secure local drive. This is an open-labeled study, and safety and feasibility are our secondary aims. Because the investigators are not blinded, on a monthly basis, the principal investigator and other study personnel will meet to monitor and evaluate enrollment, data collection, and adverse events and determine whether changes to the study protocol are required based on this information. The study undergoes full audit annually.

## Trial Status

The recruitment was initiated on November 22, 2022, with the approximate recruitment completion date in August 2024.

## Supplementary Material

SPIRIT Checklist.

Approved Consent Form.

## Abbreviations Used

AD: autonomic dysreflexia
ANS: autonomic nervous system
BP: blood pressure
ECG: electrocardiogram
EMG: electromyography
eSCS: epidural spinal cord stimulation
HR: heart rate
JJP VAMC: James J. Peters VA Medical Center
OH: orthostatic hypotension
Rr: respiratory rate
SBP: systolic blood pressure
SCI: spinal cord injury
SCS: spinal cord stimulation
tSCS: transcutaneous spinal cord stimulation
WHO: World Health Organization
