# Supplementary material for: Transcutaneous Spinal Cord Stimulation to Stabilize Seated Systolic Blood Pressure in Persons With Chronic Spinal Cord Injury: Protocol Development
Source: Neurotrauma Rep. 2023 Dec 19;4(1):838–47. doi: 10.1089/neur.2023.0063 (PMC10754346; doi:10.1089/neur.2023.0063)
Supplement: Supplemental data [file Consent.pdf]

**Version Date: August 7, 2023**	**Page: 1 of 11**
**Subject Name:**	**Informed Consent Date:**
**Principal Investigators: Dr. Caitlyn G. Peters, PhD**	**VAMC: James J Peters**
**Protocol #: 1656566**<br>**Title of Study: Targeted Transcutaneous Spinal Cord Stimulation to Restore Autonomic Cardiovascular Health in Individuals with Spinal Cord Injury**	

INTRODUCTION

You are being asked to participate in a research study that is being performed at JJPVAMC. Before you decide to take part, it is important for you to know why the research is being done and what it will involve. This includes any potential risks to you, as well as any potential benefits you might receive.

Please read the information below closely, and discuss it with your family and friends if you wish. You can also ask one of the study staff members if there is anything that is not clear to you or if you would like more details. Please take your time to decide. If you do decide to take part in this study, your signature on this consent form will show that you received all of the information below, and that you were able to discuss any questions and concerns you had with a member of the study team.

1. Purpose of study and how long it will last:

You are being asked to participate in the study because you have a spinal cord injury (SCI) and are above the age of 18 years old. The purpose of this research study is to identify an individualized map using transcutaneous spinal cord stimulation to increase blood pressure, and then compare heart rate, blood pressure, blood flow in the brain and body, and blood levels of certain hormones during a tilt with and without stimulation. The results will help guide experimental studies aimed at improving health and longevity in the SCI population. If you choose to participate in this study, and if you qualify, there will be up to an additional 14 study visits that take between 2-4 hours each. A copy of this form will be given to you and a copy will be kept by the JJPVAMC research team. This study is sponsored and funded by the VA Rehabilitation Research and Development Service.

Participants:

You will be one of about 14 participants in this study participating at JJPVAMC.

Inclusions:

- You are above the age of 18 years old;
- Traumatic spinal cord injury at T6 or above;
- Duration of injury more than 1 year;
- Wheelchair dependent;
- American Spinal Injury Association (AIS) A, B, or C
- Non-ventilator;
- Low blood pressure (males: systolic blood pressure less than 110 mmHg and/or diastolic blood pressure less than 70 mmHg; females: systolic blood pressure less than 100 mmHg and/or diastolic blood pressure less than 70 mmHg); which will be determined before testing

on each study visit.

Exclusions: If any of the following statements apply to you, you need only tell the researcher that one or more of the statements pertain to you. To ensure your privacy and confidentiality, you need not reveal which of the statements apply to you. If you choose to tell the investigator which of the statements apply to you, the information will be kept strictly confidential. You will inform the researcher if any of the following statements apply to you.

- You currently have illness or infection;
- You are pregnant
- You have controlled or uncontrolled diabetes mellitus;
- You have a neurological condition other than SCI (Alzheimer's disease, dementia, stroke, multiple sclerosis, Parkinson's disease, etc.);
- You have history of cardiovascular disease (coronary artery disease, congestive heart failure, peripheral artery disease);
- You have present or history of thrombosis (blood clot) in the last 12 months;

2. Description of the Study Including Procedures to be Used:

If you consent to participate in this research study, you will visit the research center in Suite 7A-13 of the JJPVAMC main hospital building located in the Bronx, NY for a minimum of 1 and a maximum of 15 visits. On each day of testing, please report to the laboratory, located in the SCI Research Department at JJPVAMC. You will be asked to not drink alcohol and caffeine for 12 hours prior to each study visit.

<u>**Spinal Stimulation Parameter Mapping**</u>

After you consent and confirming you have low blood pressure, you will remain seated in your wheelchair for all of the spinal stimulation parameter mapping visits. You will undergo between 1 to 12 spinal stimulation mapping study visits.

- <u>Transcutaneous spinal cord stimulation:</u> electrodes will be placed on the spinal cord at one of four different locations (T7/8, T9/10, T11/12 & L1/2). Each visit we will test two different electrode placement sites in a randomized order with at least 10 minutes in between each site. The stimulation frequency will either be set at 30 or 60 Hz, pulse width will be between 80-1000 μs, with and without a carrier frequency of 10,000 Hz, and we will use either a biphasic or monophasic waveform. Amplitude (intensity) ramping will

start at 0 mA and will be increased at intervals between 1 and 10 mA until one of the following occur: 1) systolic blood pressure is between 110-120 mmHg for males or 100-120 mmHg for females, 2) a maximum amplitude of 120 mA is reached or 3) you become uncomfortable and ask that we turn the stimulation off. Once your systolic blood pressure reaches the desired level, we will maintain the stimulation parameters will be held for 10 minutes while we continuously monitor your blood pressure, respiratory rate, pulse oxygen levels, and heart rate. We anticipate that you will undergo between 1 and 10 mapping sessions.

- Blood pressure: Brachial blood pressure will be monitored at 1 minute intervals using a standard adult blood pressure cuff on your right upper arm. In addition, beat-to-beat blood pressure will be continuously monitored on your left upper arm and finger. Blood pressure will be collected for 5 minutes before each mapping session, continuously during each session and 5 minutes after the stimulation is turned off to make sure your blood pressure returns back to baseline.
- Heart rate and respiration rate: 3 electrodes will be affixed to the chest and abdomen for continuous monitoring of heart rate and respiration rate.
- Pulse oxygen levels: The pulse oximeter will be placed on one of your fingers on your left hand to measure how much oxygen is in your blood.
- Autonomic Dysreflexia (AD) Likert Scale: Questions will be asked every 5-minutes during each mapping session to report any symptoms of AD including sweating, heart palpitations, anxiety, goosebumps, and headaches. The scale will be 0=no symptoms to 10=very bad symptoms.
- Pain Likert Scale: Questions will be asked every 5-minutes during each mapping session to report if you are experiencing any pain. The scale will be 0=no symptoms to 10=very bad symptoms.
- Skin check: Before and after each mapping session, the skin under and surrounding the electrode will be checked for any skin irritations, burns or discoloration.

Orthostatic Challenge with Stimulation and Sham-Controlled Stimulation

- Baseline Assessments: We will monitor your blood pressure, respiration rate, heart rate, pulse oxygen level and brain blood flow for 5 minutes at baseline while you rest quietly in the seated position in your wheelchair.
- Supine Assessments: Following baseline collection, you will be transferred to a tilt table to lay down. You will rest quietly for 10 minutes and then we will collect blood pressure,

respiratory rate, heart rate, pulse oxygen level and brain blood flow for 5 minutes. In the supine position, arterial stiffness will be measured, and blood will be drawn.

- Head-up Tilt Maneuver: You will be tilted to 60 degrees for 30 minutes while blood pressure, respiratory rate, heart rate and brain blood flow are continuously recorded. Blood will be drawn at 3 -, 10- and 25-minutes during the tilt. In addition, we will ask you questions about any symptoms you are feeling. If at any point, you feel unwell being upright during the tilt, the investigator will bring you down to the supine position.
- Blinded, Randomized Stimulation: You will complete the tilt study with stimulation (active) or with a sham-control stimulation (sham). The order of the visits will be randomized. On both visits, stimulation electrodes will be placed at the site where the stimulation parameters increased your systolic blood pressure was between 110-120 mmHg for males; 100-120 mmHg for females during the mapping sessions.
- Active Transcutaneous Spinal Cord Stimulation: Stimulation will be turned on at the start of the tilt and will be maintained constant throughout the tilt. It is possible that during the tilt, amplitude may be increased slightly higher than what was needed in the seated position in order to increase and maintain systolic blood pressure. If this is the case, there will be a second orthostatic challenge with active stimulation (visit 15).
- Sham-Controlled Stimulation: During the sham-control stimulation visit, the electrodes will still be placed at the optimal site. The stimulation will be briefly (30 seconds) ramped up to the same level as active stimulation, then ramped down and turned off for the remainder of the tilt. The stimulator and electrodes will remain attached to you during the whole study visit, similar to the active stimulation.
- Blood pressure: Brachial blood pressure will be monitored at 1 minute intervals using a standard adult blood pressure cuff on your right upper arm. In addition, beat-to-beat blood pressure will be continuously monitored on your left upper arm and finger. Blood pressure will be collected for 5 minutes in the seated position, in the supine position and continuously throughout the tilt session.
- Heart rate and respiration rate: 3 electrodes will be affixed to the chest and abdomen for continuous monitoring of heart rate and respiration rate. Heart rate and respiration rate will be collected for 5 minutes in the seated and supine position, and continuously throughout the tilt session.
- Brain blood flow: a small Doppler ultrasound probe with ultrasound gel will be placed over your left temple to measure blood flow velocity through an artery in your brain (the middle

cerebral artery) at rest and during the orthostatic tilt. Brain blood flow will be collected for 5 minutes in the seated and supine position, and continuously throughout the tilt session.

- Arterial stiffness: stiffness of your arteries will be assessed by placing a small pencil like probe against your neck at the carotid artery and against your thigh at the femoral artery to calculate the difference in time it takes for the pulse to get to each artery. Arterial stiffness will be measured during the rest period while you are lying down.
- Blood Draws: Blood will be drawn from a vein in your arm or hand over the course of the study visit. Each blood collection tube will contain about 1.2 teaspoons (6mL) of your blood. Three blood samples will be taken during the lying down position and at 60 ° tilt at 3, 10 and 25 minutes. A certified and trained researcher will gently insert a very small needle into the vein to collect blood. Once the blood has been collected, the needle will be removed and the site where blood was drawn will be cleaned and covered.
- Pulse oxygen levels: The pulse oximeter will be placed on one of your fingers on your left hand to measure how much oxygen is in your blood.
- Orthostatic Hypotension (OH) Likert Scale: Questions will be asked every 5-minutes during the active and sham stimulation tilt to report any symptoms of OH including dizziness, light headedness, blurred vision, nausea, weakness, confusion, fatigue, and passing out. The scale will be 0=no symptoms to 10=very bad symptoms.
- Autonomic Dysreflexia (AD) Likert Scale: Questions will be asked every 5-minutes during the active and sham stimulation tilt to report any symptoms of AD including sweating, heart palpitations, anxiety, goosebumps, and headaches. The scale will be 0=no symptoms to 10=very bad symptoms.
- Pain Likert Scale: Questions will be asked every 5-minutes during the active and sham stimulation tilt to report if you are experiencing any pain. The scale will be 0=no symptoms to 10=very bad symptoms.
- Skin check: Before and after each tilt session, the skin under and surrounding the electrode will be checked for any skin irritations, burns or discoloration.

While you are being tested in this study, investigators may identify test results that they believe should be reviewed by your primary care doctor. If that happens, they will call you within a week of the test to let you know. If you agree, the research team will send the test results to your primary care doctor. You acknowledge that the researchers are not specifically looking for any medical problems, so it is unlikely that they will find any underlying issues. You understand that the tests for this study are not the same tests done as part of regular medical care.

JJPVAMC IRB
Effective Date: September 26, 2023

3. Description of any Procedures that may Result in Discomfort or Inconvenience:

You have been told that the study described above may involve the following risks and/or discomforts:

Heart rate and breathing rate: You may experience some discomfort when the electrodes are removed from your skin and some skin irritation at the site of electrode placement.

Blood pressure: You may experience some discomfort when the blood pressure cuffs around your upper arm and middle finger are inflated.

Brain blood flow: You may experience some discomfort when the head harness is used to secure the ultrasound probe to your head for assessment of brain blood flow.

4. Expected Risks of Study:

Heart rate and breathing rate: You may experience some skin irritation when the electrodes are removed.

Blood pressure: You may experience some tingling or numbness sensation when the blood pressure cuff around your upper arm and middle finger are inflated.

Blood draw: You may feel discomfort when the needle is being placed in your arm vein. You may experience bruising or infection at the site of skin puncture, temporary faintness and rarely temporary loss of consciousness.

Orthostatic tilt: You may feel dizzy, light-headed or nauseous as the research staff passively repositions you from lying down to 60-degree tilt. A staff member will be supporting you during this tilt, but if you feel uncomfortable, you can stop the test at any time.

Transcutaneous spinal cord stimulation: You may get headaches, autonomic dysreflexia, pain, severe fractures and muscle tears using the transcutaneous stimulation. The electrical stimulation may cause skin irritation and burns. We are using protective electrodes to minimize the risk of skin irritation and burns. If any of these potential risks occur, the stimulation will cease immediately, and the investigators will monitor the situation.

JJPVAMC IRB
Effective Date: September 26, 2023

As with any research, there may be unforeseen risks and discomforts. Dr. Harel, a medical doctor associated with the study, will be available and present during testing to for clinical intervention if necessary or to treat any medical emergency.

5. Expected Benefits of the Study

There may be no direct benefit to you from this study, but any information that the researchers get from this study may help others.

6. Other Treatments Available:

Participation in the study is voluntary and the alternative to this study is to not participate and seek clinical advice from your doctor.

7. Use of Research Results:

The researchers will let you and your physician know of any significant new findings made during this study which may affect your willingness to participate in this study. All research material generated from the study will remain in the possession of Dr. Caitlyn G Peters and her study team at the JJP VAMC. Your medical records will be maintained according to this medical center's requirements and all electronic and hardcopy. All electronic and hardcopy Research Records will be retained according to National Archives and Records Administration, Records Schedule RCS-10-1.

Access to the research materials generated from the study will be restricted to Dr. Caitlyn G. Peters and her study team. If results of this study are reported in medical journals or at meetings, you will not be identified by name, by recognizable photograph, or by any other means without your specific consent. No information by which you can be identified will be released or published unless required by law. In order to comply with federal regulations, research records identifying you may be reviewed by the following:

All specimens obtained during this study will be stored in the Basic Science Laboratory at The Center on the Medical Consequences of Spinal Cord Injury located at the James J. Peters Veterans Affairs Medical Center, Bronx, NY. Samples will be stored for possible future analysis under this protocol. Once the samples are exhausted or the study is closed, the remaining samples will be destroyed in accordance with JJP VAMC policy.

□__________ By checking this box and initialing, you agree to be contacted by the Principal Investigator or her investigative team at a future date for additional studies being conducted in the Center for the Medical Consequences of SCI.

Authorized representatives of the JJPVAMC (e.g. Institutional Review Board, Research Compliance Officer) and VA, including the Office of Research Oversight (ORO), Federal Agencies such as the Government Accounting Office (GAO), VA Office of Inspector General (OIG), and Office for Human Research Protections (OHRP) may have access to your research records. If this research involves articles regulated by the FDA, the FDA may choose to inspect and copy research records that identify individual research subjects.

Clinical Trials:

A description of this clinical trial will be available on http://www.ClinicalTrials.gov. This website will not include information that can identify you. At most, the website will include a summary of the results. You can search this website at any time using the NCT# (NCT05180227) to locate this study.

8. Special Circumstances:

Your participation in this study will be included in the VHA health record. A copy of the signed informed consent form and signed HIPAA authorization for participation in the study will be in your health record. The study staff has no real or apparent conflicts of interest involving this study

9. Compensation and/or Treatment in the Event of Injury:

The VA must provide necessary medical treatment to a research subject injured by participation in a research project approved by a VA R&D Committee and conducted under the supervision of one or more VA employees. Further information about compensation and medical treatment may be obtained from the medical administration service at this VA medical center.

10. Voluntary Participation:

You are not required to take part in this study; your participation is entirely voluntary. You can refuse to participate in this study or withdraw your participation in this study after you consent without penalty or loss of VA or other benefits to which you are entitled.

11. Termination of Participation:

JJPVAMC IRB
Effective Date: September 26, 2023

You can refuse to participate now or you can withdraw from the study at any time after giving your consent. This will not interfere with your regular medical treatment, if you are a patient. The investigator also has the right to withdraw you from the study at any time for reasons including, but not limited to, medical concerns (your health and safety are in jeopardy with continued participation in the study), non-compliance (you miss several scheduled appointments without notification) and protocol deviations (exclusion/inclusion criteria change and you are no longer eligible to participate).

12. Costs and Reimbursements:

As a veteran or non-veteran, you will not be charged for any treatments or procedures that are part of this study. For veterans who are required to pay co-payments for medical care and services provided by VA, these co-payments will continue to apply for medical care and services provided by VA that are not part of this study.

You will receive $100 compensation for each study visit you participate in, with a total amount of $1500 if you complete all study visits.

13. Contact Person(s):

To obtain answers to questions about the research, report or seek treatment for a research-related injury, or to voice concerns or complaints about the research contact the following:

- **During the Day: Caitlyn Peters, PhD (718) 584-9000 x.1706**
- **After Hours: Caitlyn Peters, PhD (908) 672-8038**

I understand that should I wish to discuss my participation in this study with any other doctor or layperson, I can contact Mary Sano, Ph.D., Associate Chief of Staff (ACOS) R&D Program at the JJP VAMC by requesting an appointment at (718)741-4228 hospital extension 4228, first floor in the research building, Room 1F-01 If I have questions, concerns, and/or complaints or to offer input.

RESEARCH SUBJECTS' RIGHTS: I have read or have had read to me all of the above. Dr. Caitlyn Peters or her delegate has explained the study to me and answered all of my questions. I have been told of the risks or discomforts and possible benefits of the study. I have been told of other choices of treatment available to me.

I understand that I do not have to take part in this study, and my refusal to participate will involve no penalty or loss of rights to which I am entitled. I may withdraw from this study at any time without penalty or loss of VA or other benefits to which I am entitled.

The results of this study may be published, but my records will not be revealed unless required by law. This study has been explained to me. I have had a chance to ask questions. I voluntarily consent to participate in this study. I will receive a signed copy of this consent form.

______________________________	_______________	_________
Subject Signature	Date	Time
______________________________	_________________________	_________
Person Obtaining Informed Consent (Print Name) (Investigator or Delegate as indicated on Assurance Page)	Signature of Person Obtaining Informed Consent	Date

VERBAL CONSENT IF THE PARTICIPANT LACKS UPPER LIMB FUNCTIONS TO COMFORTABLY WRITE

________________________________ is unable to sign the consent form due to impaired arm function. I certify that I have carefully explained the purpose and nature of this research to him/her in appropriate language and he/she has had an opportunity to discuss it with me in detail. I have answered all of his/her questions and he/she has consented to participate in this research. I, therefore, am signing the consent form to document that he/she has given his/her consent to participate in this research study.

Person Obtaining Consent:

Name: ________________________________

Signature: ______________________________

Date: ________________________

Witness Name: __________________________

Signature: ______________________________

Date: ______________________

JJPVAMC IRB
Effective Date: September 26, 2023
